# The Differences between Gluten Sensitivity, Intestinal Biomarkers and Immune Biomarkers in Patients with First-Episode and Chronic Schizophrenia

**DOI:** 10.3390/jcm9113707

**Published:** 2020-11-18

**Authors:** Michał Dzikowski, Dariusz Juchnowicz, Izabela Dzikowska, Joanna Rog, Michał Próchnicki, Małgorzata Kozioł, Hanna Karakula-Juchnowicz

**Affiliations:** 1 1st Department of Psychiatry, Psychotherapy and Early Intervention, Medical University of Lublin, 20-439 Lublin, Poland; michal.nfs@wp.pl (M.D.); rog.joann@gmail.com (J.R.); michalprochnicki@gmail.com (M.P.); karakula.hanna@gmail.com (H.K.-J.); 2 Department of Psychiatric Nursing, Medical University of Lublin, 20-093 Lublin, Poland; 3 Department of Dermatology, Venerology and Paediatric Dermatology, Medical University of Lublin, 20-080 Lublin, Poland; izadzikowska.derm@gmail.com; 4 Chair and Department of Medical Microbiology, Medical University of Lublin, 20-093 Lublin, Poland; malgorzata.koziol@gmail.com

**Keywords:** schizophrenia, low-grade inflammation, gut–brain axis, gut–microbiota–brain axi, inflammation, gut permeability, gluten, IgG antibodies

## Abstract

Schizophrenia is a heterogeneous disorder without a fully elucidated etiology and mechanisms. One likely explanation for the development of schizophrenia is low-grade inflammation, possibly caused by processes in the gastrointestinal tract related to gluten sensitivity. The aims of this study were to: (1) compare levels of markers of gluten sensitivity, inflammation and gut permeability, and (2) determine associations between gluten sensitivity, inflammation, and intestinal permeability in patients with first-episode/chronic (FS/CS) schizophrenia and healthy individuals (HC). The total sample comprised 162 individuals (52 FS; 50 CS, and 60 HC). The examination included clinical variables, nutritional assessment, and serum concentrations of: high-sensitivity C-reactive protein (hs-CRP), interleukin-6 (IL-6), soluble CD14 (sCD14), anti-*Saccharomyces cerevisiae* antibody (ASCA), antigliadin antibodies (AGA) IgA/IgG, antibodies against tissue transglutaminase 2 (anti-tTG) IgA, anti-deamidated gliadin peptides (anti-DGP) IgG. A significant difference between groups was found in sCD14, ASCA, hs-CRP, IL-6 and AGA IgA levels. AGA IgG/IgA levels were higher in the FS (11.54%; 30.77%) and CS (26%; 20%) groups compared to HC. The association between intestinal permeability and inflammation in the schizophrenic patients only was noted. The risk for developing schizophrenia was odds ratio (OR) = 4.35 (95% confidence interval (CI 1.23–15.39) for AGA IgA and 3.08 (95% CI 1.19–7.99) for positive AGA IgG. Inflammation and food hypersensitivity reactions initiated by increased intestinal permeability may contribute to the pathophysiology of schizophrenia. The immune response to gluten in FS differs from that found in CS.

## 1. Introduction

Despite more than 100 years of research into schizophrenia, its etiology has still not been fully elucidated. This is due to the heterogeneity of its etiopathogenesis and the clinical course [1]. There are many overlapping and complementary theories and hypotheses about the causes of the disorder. Its development is probably due to the interaction of genetic and environmental factors that lead to neurostructural, neurochemical, and neurofunctional changes in the brain [2,3,4,5]. In 1992, Smith presented the macrophage (cytokine) theory of schizophrenia, according to which the disorder is based on immune–inflammatory reactions. The author indicated the gastrointestinal tract as an area where the causes of immune activation should be searched for [6]. More and more evidence, both from model and clinical studies, confirms the validity of Smith’s theory, with the highest agreement on increased levels of pro-inflammatory cytokines interleukin-1β (IL-1β), interleukin-6 (IL-6), interleukin-10 (IL-10), tumour necrosis factor α (TNF-α) [7,8]. Moreover, in 2020, Roomruangwong et al. proposed a model in which the key factor leading to the development of numerous neuroimmune disorders is the compensatory immune-regulatory reflex system (CIRS). According to these researchers, CIRS negatively regulates the immune–inflammatory response system, leading to a new homeostatic setpoint between both components and changes in immune response [9].

In terms of the importance of the proper functioning of the gastrointestinal (GI) tract for the development of mental disorders, the gut–microbiota–brain axis, which is bidirectional communication between the GI tract and the brain, plays a significant role. The interactions take place via endocrine, metabolic, neuronal, and immune pathways [10,11,12,13].

Disturbances in the balance of the brain–gut–microbiota axis may result from the dysfunction of the intestinal barrier integrity, which is related to the activity of modulators that ensure its proper structure (including occludins and the endogenous protease called zonulin). Intestinal hyperpermeability causes the penetration of antigens into the circulatory system and the activation of the immune–inflammatory cascade [14,15,16]. Evidence confirming the existence of structural damage to the intestinal barrier in people with schizophrenia comes from, among others, autopsy studies or studies on the markers of enteritis and the translocation of bacteria [17,18,19,20].

A nutritional factor that may contribute to the immune–inflammatory activation in the lumen of the GI tract is gluten, which is a component of grains such as wheat and barley. As early as in the 1950s, attention was drawn to the relationship between the occurrence of juvenile schizophrenia and celiac disease [19], currently considered to be a gluten-dependent autoimmune enteropathy of the small intestine [20,21]. In the 1960s, Dohan showed a relationship between the risk of developing schizophrenia and the amount of cereals consumed [22].

Studies conducted in recent years indicate a higher prevalence of clinically significant anti-gluten antibody titers (AGA IgA and AGA IgG) among patients with schizophrenia compared to healthy individuals [23,24,25,26,27,28]. This relationship seems to also be confirmed by the clinical cases which describe a partial or complete remission of schizophrenia symptoms after following a gluten-free diet [29,30,31] and the results of the first randomized, double-blind clinical study [32]. The data on the prevalence of positive results and differences in the concentration of anti-gluten antibodies among patients in the first episode of schizophrenia remain inconsistent. There is also a lack of research to explain and link potential mechanisms related to the immune response to gluten proteins, the integrity of the intestinal barrier, and the activation of the inflammatory cascade in schizophrenia.

The aim of this study was to determine the differences in the concentrations of markers related to IgG and IgA sensitivity, inflammation and gut integrity between the first episode of schizophrenia (FS), chronic schizophrenia (CS) patients, and healthy individuals (HC), and to establish potential connections between inflammation, gluten sensitivity and intestinal permeability.

## 2. Materials and Methods

The study included 162 participants: 102 patients that met the criteria of schizophrenia (SZ) according to the Diagnostic and Statistical Manual of Mental Disorders, Fifth Edition (DSM-5) [33] and 60 healthy individuals (HC) as a control group. Among the SZ group, 52 were first episode (FS), and 50 were chronic patients (CS). The exclusion criteria were: co-occurrence of neurological diseases, mental retardation, organic brain dysfunction, addiction (except nicotine and caffeine), unstable phase of other somatic condition, celiac disease and other autoimmune diseases, or clinical signs of inflammation (high-sensitivity C-reactive protein; hsCRP > 5 microgram/milliliter) and/or leukocytosis (10,000 10^3^/microliter) (present during admission to the study).

The study was conducted in accordance with the Declaration of Helsinki, and the protocol was approved by the Ethics Committee of the Medical University of Lublin, Poland (project identification code: KE-0254/231/2013).

### 2.1. Blood Collection

Venous blood (20 mL) samples were collected after overnight fasting. Serum was obtained by centrifugation (at 2000× g, 10 min at room temperature) and was stored at −80 °C until further analysis.

### 2.2. Laboratory Tests

The markers of immune reactivity to gluten, inflammation, and intestinal permeability were tested by the enzyme-linked immunosorbent assay (ELISA) method using following kits: Gliadin IgA (Demeditec Diagnostics GmbH); Gliadin IgG (Demeditec Diagnostics GmbH), Transglutaminase IgA ELISA (BlueWell); Deamidated Gliadin IgG ELISA (BlueWell); Human Interleukin-6 ELISA Kit II (Becton, Dickinson and Company BD Biosciences); high-sensitivity C-reactive protein ELISA (Demeditec Diagnostics GmbH); Human Anti-*Saccharomyces cerevisiae* Antibody (ASCA) (BlueGene); and Human soluble CD14 Quantikine ELISA (R&D Systems). All analyses were performed according to the manufacturer’s instructions.

The cut-off points of elevated antibodies were estimated for AGA IgG/IgA, anti-DGP IgG and anti-tTG IgA according to the manufacturer’s instructions, and for hs-CRP [34] IL-6 [35], sCD14 [36], ASCA [37] based on earlier studies.

### 2.3. Sociodemographic and Clinical Data

Characteristics of the study sample were determined using a self-constructed questionnaire including sociodemographic/lifestyle information. The clinical data on patients were obtained from a supervising physician. The doses of antipsychotic medication were calculated based on defined daily doses (DDDs) to 1 mg olanzapine [38].

The severity of gastrointestinal symptoms was examined based on the Visual Analogue Scale for Irritable Bowel Syndrome (VAS-IBS) [39].

The severity of schizophrenia symptoms was assessed by a well-trained physician (M.D.) using the Polish adaptation of the Positive and Negative Symptom Scale (PANSS) [40]. The examination was performed twice: during the blood collection and at the time of the hospital discharge.

### 2.4. Gluten Intake

Dietary assessment was performed as previously described [15] using the Food Frequency Questionnaire with six answers (FFQ-6), which included the main sources of gluten in the Polish diet. Briefly, the assessment was performed by a clinical dietitian with experience in conducting nutritional interviews (J.R.). The participants described the frequency and amount of food intake, and to determine a portion size, an “Album of photographs of food products and dishes” was used [41]. The amount of gluten was estimated by multiplying protein containing gluten cereal by 0.8, as in earlier studies [42,43]. Gluten in the diet was expressed in grams per day per person.

### 2.5. Statistical Analysis

Descriptive statistics were presented using the mean value, median and standard deviation, and for the categorical variables, using percentage. The Shapiro–Wilk test was performed for the distribution of the variables explored. The differences between groups in categorical variables were examined with the chi^2^ test. To determine differences between the examined groups, t-student or Mann–Whitney U test (in case of two groups), and ANOVA analysis of variance or Kruskal–Wallis one-way analysis of variance (in case of three groups) were used. To determine the relationship between examined variables, the Spearman’s rho correlation, logistic regression analysis with the quotient of the odds ratio, and analysis of covariance ANCOVA test were used. The value of *p* < 0.05 was considered statistically significant. To further analyze variables that significantly correlated with inflammation, stepwise multiple regression analysis was applied. The value of *p* < 0.05 was considered statistically significant. In case of multiple comparisons, Bonferroni correction was applied. Statistical analyses were conducted using Statistica 9.1 software (TIBCO Software Inc., Palo Alto, CA, USA).

## 3. Results

### 3.1. Characteristics of the Examined Sample

The study sample consisted of 162 participants aged 18 to 65 years: 52 patients in the first episode of schizophrenia (FS group), 50 patients with chronic schizophrenia (CS group) and 60 healthy individuals as a control group (HC group). The socio-demographic and clinical characteristics of the examined group are shown in Table 1. There was a similar proportion of female and male participants in the examined groups (55.8%, 52%, and 41.7% of males in FS, CS and HC group, respectively). Significant differences between the examined groups included body mass index (BMI) (*p <* 0.001), gluten intake (*p* = 0.004) and gastrointestinal (GI) complaints (*p* = 0.002). The CS group had the highest BMI and the FS group the lowest BMI. The HC group had a lower intake of gluten compared to the CS group. The greatest severity of gastric symptoms was the HC group and the least GI manifestations were reported by the CS group.

The patient groups (FS and CS) differed in the clinical picture: duration of illness, number of hospitalizations, doses of olanzapine equivalents and severity of psychopathological symptoms (see: Table 1). The severity of the psychopathological symptoms was higher in the FS group (mean ($\bar{x}$) = 95.85; median (Me = 100) than in the CS group ($\bar{x}$ = 73.66; Me = 73.5).

### 3.2. The Differences in Gluten Sensitivity and Inflammation Markers between Patients with Schizophrenia and Healthy Individuals

The differences in gluten sensitivity and inflammation markers between the examined groups are shown in Table 2. The concentrations of AGA IgA antibodies were higher in the CS than HC group (*p =* 0.038) and the concentrations of AGA IgG antibodies did not differ between the patients and healthy individuals (*p >* 0.05). There were no significant differences between the three groups in terms of other markers related to an abnormal reaction to gluten (anti-tTG IgA and anti-DGP IgG) (*p* > 0.05).

With Bonferroni adjustment, HCs exhibited a higher concentration of ASCA than the FS group (*p* < 0.017). sCD14 concentration differed between the HC and CS group—the patients presented with more severe intestinal inflammation with expression of this marker (*p* < 0.001).

The greatest inflammatory state was found in the CS group, reflected by the highest concentration of hsCRP compared with both the HC group and FS group (*p* < 0.001) and IL-6 compared with the HC group (*p* = 0.022). The differences in IL-6 (between FS and HC) and AGA IgG concentrations were statistically insignificant after Bonferroni correction *(p* > 0.017).

### 3.3. The Prevalence of Clinical Relevance

An elevated titer of AGA IgA antibodies was found in 11.54% of FS, 26% of CS and 5% of HC participants, respectively (see Table 3). Elevated concentrations of AGA IgG were also more frequent in the patient groups compared to healthy individuals (30.77% of FS and 24% of CS group compared to 10% of HS). The clinically significant titers of anti-tTG IgA and anti-DGP were detected only in a small percentage of all the examined groups (anti-tTG IgA: 1.92%, 2%, 0% of FS, CS, HC groups, respectively, and anti-DGP: 5.77%, 2% of FS and CS groups, respectively, and none in the HC group).

High concentrations of both intestinal-related markers (ASCA and sCD14) were found only in the CS group (6% and 4%, respectively). In the FS, CS, and HC groups, 17.31%, 36% and 6.67% had elevated serum concentrations of hsCRP and 84.62%, 90% and 91.67% had elevated serum concentrations of IL-6, respectively.

### 3.4. The Relationship between Gluten Sensitivity and Inflammation

We found a positive association between AGA IgG and ASCA blood concentrations in the patient groups (Spearman’s rank correlation coefficient (r)) = 0.294; *p* < 0.05 in CS and r = 0.034; *p < 0.05* in the FS group) (see Supplementary Materials Table S1). A relationship between AGA IgG and AGA IgA antibodies was detected only in the FS group (r = 0.299; *p* < 0.05).

We did not find a correlation between the markers of gluten sensitivity (tTG2 IgA and DGP IgG) and inflammation (*p* > 0.05).

hsCRP was positively associated with sCD14 blood concentration in the patient groups (FS: r = 0.381; *p* < 0.05 and CS: r = 0.299; *p* < 0.05).

### 3.5. The Relationship between Gluten Sensitivity, Inflammation and a Phase of Schizophrenia

In the FS and CS groups, there was a negative relationship between AGA serum concentrations and the duration of illness (AGA IgA in FS: r = −0.289; *p* < 0.05 and AGA IgG in CS: r = −0.394; *p* < 0.005).

In the CS group, intestinal inflammation (ASCA) was negatively associated with the duration of illness (r = −0.387; *p* = 0.05).

In the FS group, IL-6 was positively associated with the duration of illness (r = 0.310; *p* < 0.05).

The dose of olanzapine equivalents was connected with the duration of illness (r = 0.480; *p <* 0.05), age (r = 0.331; *p* < 0.05), and BMI (r = 0.324; *p* = 0.05), while the severity of gastrointestinal symptoms was positively related to age (r = 0.331 *p* < 0.05) in the FS group. In HCs, higher BMI was related to a greater number of cigarettes smoked per day (r = 0.384; *p* < 0.05).

### 3.6. The Relationship between Gluten Sensitivity, Inflammation and Lifestyle Factors

We found no correlation between daily gluten intake and markers of gluten response or inflammation across the examined groups (*p* > 0.05).

There was a relationship between AGA IgG serum concentrations and: the severity of gastrointestinal symptoms in the FS group (r = 0.322; *p* < 0.05) and age in the CS group (r = −0.419; *p <* 0.05) (see Supplementary Materials Table S2). In the CS group, anti-tTG concentration was negatively related to the number of meals per day (r = −0.320; *p* < 0.05).

In the CS group, intestinal inflammation (ASCA) was inversely associated with age (r = −0.401; *p* < 0.05).

In the FS group, IL-6 was negatively associated with the number of cigarettes smoked per day (r = −0.368; *p* < 0.05). In the FS group, hsCRP blood concentration was positively associated with age (r = 0.276; *p* < 0.05), and in the CS and the HC groups with BMI (r = 0.412; *p* < 0.05 and r = 0.560; *p* < 0.05, respectively). In HCs, hsCRP blood concentration was related to the number of cigarettes smoked per day (r = 0.379; *p* < 0.05) and the severity of gastrointestinal symptoms (r = 0.275; *p* < 0.05).

In the HC group, inflammation was positively associated with age (IL-6: r = 0.278; *p* < 0.05; hsCRP: r = 0.387; *p* < 0.05).

### 3.7. The Effect of Inflammation and Gluten Sensitivity on the Risk of Schizophrenia

To determine risk factors for developing schizophrenia, logistic regression analysis was performed. The increased AGA IgG (≥ 12 U/mL) antibody titer was related to a four-fold higher ODDS of schizophrenia development odds ratio (OR) = 4.00 (95% confidence interval (CI)): 1.43–11.19)) in the FS group and almost a seven-fold higher ODDS (OR = 6.68 (95% CI: 1.71–25.04)) in the CS group.

In the CS group, an increase of one unit of sCD14 was connected with an increased risk of schizophrenia development by 1.002 (95% CI: 1.001–1.003), and an increase of one unit of hsCRP was associated with an increased risk of schizophrenia development by 1.486 (95% CI: 1.19–1.86).

### 3.8. Potential Risk Factors for Inflammation

To assess risk factors for inflammation, ten predictor variables (risk factors), namely AGA IgA and IgG, sCD14, ASCA, IL-6, daily gluten intake, BMI, age, the severity of gastric symptoms, the number of cigarettes smoked per day, and olanzapine equivalents dose, were considered in a risk factor model. A stepwise multiple regression analysis was performed to determine significant associations.

In the FS group, sCD14 blood concentration was independently associated with CRP and accounted for 10% of CRP variability (*p* < 0.05). The number of cigarettes smoked per day was related to IL-6 blood concentration and explained 10% of its variability (*p* < 0.05).

In the CS group, IL-6 and BMI were related to CRP blood concentration. This model accounted for 35% of CRP variability (*p* < 0.05). sCD14 and ASCA were independently associated with IL-6 and predicted 31% of IL-6 variability. However, the model which included hsCRP and sCD14 accounted for 35% of IL-6 variability (*p* < 0.05).

## 4. Discussion

The connection between gut-related immune responses, inflammation and gluten sensitivity in schizophrenia needs clarification. Therefore, the aim of our study was to find connections between markers of gluten sensitivity, inflammation and gut permeability in the first episode of schizophrenia (FS) and chronic schizophrenia (CS), and determine differences in immune responses to gluten between healthy individuals and patients suffering from schizophrenia. The obtained results indicate changes in immune–inflammatory pathways related to gluten sensitivity in schizophrenia.

We have shown that the prevalence of increased AGA antibody titers is significantly higher in the patient groups compared to healthy subjects: 11.54% in the FS group and 26% in the CS group (*p* = 0.005) compared to 5% in the HC group for AGA IgA, and 30.77% in the FS group and 20% in the CS group compared to 10% in the HC group for AGA IgG (*p* = 0.023). In the studies conducted so far, increased levels of AGA antibodies have been observed in approximately 30% of patients with schizophrenia [25,29], but with clinically significant results ranging from 1.43% to 59%, which may be due to the heterogeneity of the groups [21,22,23,25,29]. However, the majority of observations did not take into account the stage of the disease [24,25,26,28], which appears to be a strong determinant of the results obtained.

Most likely, with increasing duration of the disease, there is a conversion in the number of gliadin antibodies produced (from the subclass IgG to IgA). This is confirmed by the inverse relationship observed by us between AGA IgG antibodies and age and duration of the disease. These results are partly in line with literature data [23], but the mechanisms associated with the change in the immune response to gluten proteins remain unclear.

In some studies, no differences in antibody titer against gluten components were shown between healthy individuals and schizophrenia patients [44,45]. However, despite the unchanged immune response of patients, Severance et al. observed a relationship between the level of antibodies to gluten in blood serum and their level in cerebrospinal fluid [44].

In our study, elevated AGA antibody titers increased the risk of developing schizophrenia by about four to seven times (for FS: AGA IgG OR = 4.000 (95% CI 1.43–11.189); for CS: 6.676 (95% CI 1.708–25.035)), which is consistent with a meta-analysis carried out by Lachance and McKenzie [46].

Compared to healthy subjects, chronic patients had significantly higher concentrations of AGA IgA antibodies (*p* = 0.038). To the best of our knowledge, only one study has assessed the differences in levels of antibodies between first episode schizophrenia (FES) and chronic patients [47], showing differences in AGA IgG and IgA antibody classes in patients in the first episode of the disease and AGA IgG antibodies in chronic patients compared to healthy subjects. This may, at least in part, explain discrepancies in the literature about gluten antibodies in patients with psychotic disorders. In studies not taking the duration of the disease into account, a large majority confirm the existence of a higher concentration of at least one subclass of AGA [27,32,48].

Significantly higher levels of two inflammatory markers, namely hsCRP and IL-6, were found in the CS group (compared to the HC and FS groups, and the HC group, respectively), confirming that chronic, persistent low-grade inflammation is likely to occur as disease duration increases. In addition, a negative relationship between IL-6 and the number of cigarettes smoked per day was found in the FS group (r = −0.368; *p* < 0.05), which may be related to the partial anti-inflammatory properties of cotinine, as confirmed by the linear regression model for IL-6 in the FS group, where the number of cigarettes smoked was an important predictor [46].

So far, there is no clear answer as to whether the level of inflammatory markers changes as the disease progresses. Significantly higher levels were noted in both those in the first episode of disease [47] and those with chronic illness (the average duration of the disease in the study by Rowland et al. was over 25 years) [48].

In contrast to the studies carried out so far [17,25], we found higher ASCA levels among healthy subjects compared to patients with schizophrenia. However, the marker concentration of enterocolitis was associated with AGA IgG values only among patients (FS r = 0.294; *p =* 0.034 and CS r = 0.315; *p =* 0.026), and the elevated concentration of ASCA was found only in chronic patients. The altered immune response to gluten and its relationship to the maintenance and/or initiation of low-grade inflammation may occur only in the predisposed population [17]. This is indicated by the positive correlation between sCD14 and hsCRP observed only in the subgroups of patients (FS: r = 0.381; *p =* 0.005 and CS: r = 0.299; *p* = 0.035). Chronic low-grade inflammation sustained by the overproduction of gluten antibodies may, in predisposed individuals, lead to activation of the immune–inflammatory cascade [14,49]. This hypothesis is partially confirmed by a study carried out in newborn babies. Untreated maternal coeliac disease is linked with a higher risk of non-affective psychosis in the offspring. Maternal exposure to IgG AGA antibodies and their levels above the 90th percentile in a dried blood spot during the newborn period was linked with a higher risk of schizophrenia development in the future. The antibodies detected in the blood of children were predominantly derived from the maternal circulation and probably represented maternal reactivity to gluten [50].

A higher concentration of sCD14 was observed in the CS group compared to the HC group (*p <* 0.001), which is consistent with data provided by other authors [51,52]. sCD14 is not only a marker of bacterial translocation, but also reflects the overall activation of monocytes (including bacterial ligands) [51,53]. Weber et al. have shown that the concentration of sCD14 in people with schizophrenia compared to control groups is significantly higher many years before the onset of the first symptoms of the disease, while there is no difference in the concentration of markers of inflammation such as hsCRP. This suggests a certain dynamic in the changes concerning sCD14 and CRP that is characteristic of the schizophrenic process. The increased concentration of sCD14 precedes the appearance of the first clinical symptoms of schizophrenia, which appear as a manifestation of a strong pro- and anti-inflammatory imbalance [52]. This is confirmed by our logistic regression analysis, which showed that sCD14 is a predictor of observed inflammation (hsCRP and IL-6).

**Figure 1:**
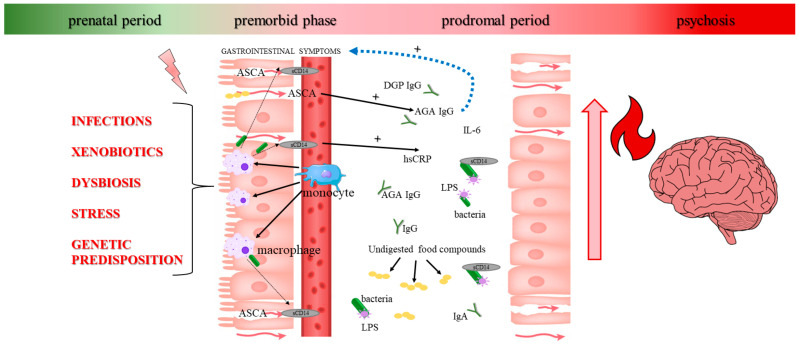
The interplay between genetic susceptibility and environmental factors across prenatal and early life may lead to intestinal hyperpermeability via immune activation. Through the production of intestine-related proteins (sCD14 and ASCA), undigested gluten-derived compounds trigger an immune–inflammatory response (secretion of AGA and CRP) disproportionate to the amount of gluten intake. High levels of IgG AGA antibodies provoke gastrointestinal manifestations. There is a conversion of gliadin antibodies produced (from the subclass IgG to IgA) during the disease course. Abbreviations: hs-CRP—high-sensitivity C-reactive protein, IL-6—interleukin-6, sCD14—soluble CD14, ASCA—anti-*Saccharomyces cerevisiae* antibody, AGA—antigliadin antibodies, DGP—deamidated gliadin peptides; LPS—lipopolysaccharides.

The existence of a relationship between inflammation and the intestinal barrier only among patients suggests that immune processes associated with brain–gut–microbiota dysfunction are involved in the development and/or maintenance of schizophrenia symptoms, and anti-psychotic treatment may strongly modulate this response [13]. It seems to be necessary to identify substances modulating the gut–microbiota–brain axis (see Figure 1). Some observations suggest that second-generation drugs can modify the composition of the intestinal ecosystem [54].

The figure below depicts a possible relationship between gluten sensitivity and schizophrenia based on the results obtained in this study and hypotheses proposed in previous papers.

In addition, as shown by the results described above, there are numerous differences in the immune response to gluten proteins between patients in the first episode of the disease and those who are chronically ill. To better understand the importance of immunological processes and the gut–brain axis in the development of schizophrenia and methods for its prevention, further studies should take into consideration the duration of the disease, people in high-risk groups, and people predisposed to the development of psychotic disorders. The results of an interventional pilot study that applied an elimination (gluten-free) diet in patients with schizophrenia are promising [32]. Confirmation of the results within a larger group, in practical terms, would allow a more personalized form of co-therapy of psychotic disorders.

### Limitations

The study has some potential limitations. First, the study group was relatively small, and most patients were treated with antipsychotic drugs. Medication could affect gut microbiota (a key indicator of intestinal integrity) and inflammation. Another limitation was that no other markers for gut integrity and inflammation were measured. The concentration of examined variables in peripheral blood is not always related to their level in the cerebrospinal fluid. Further studies should include other variables (e.g., zonulin, occludin, other interleukins), both in blood and cerebrospinal fluid. Given the essential role of the gut ecosystem in processes involved in gut integrity, immune response, and pro/anti-inflammatory homeostasis, microbiota assessment is necessary to determine the role of gut–brain dysfunction in schizophrenia.

## 5. Conclusions

1. The study indicates differences in markers of intestinal permeability, inflammation, and gluten sensitivity between patients suffering from schizophrenia and healthy individuals.
2. The immune response to gluten noted in schizophrenia patients depends on the phase and duration of illness.
3. The connection between inflammation, intestinal-related markers, and gluten sensitivity only in the patient groups indicates possible gut–microbiota–brain axis disruptions in schizophrenia.
4. Further studies are needed to confirm the role of immune–inflammatory pathways, the gut–microbiota–brain axis, and gluten sensitivity in the pathophysiology of schizophrenia.

## Figures and Tables

**Table 1:** Characteristics of participants

Group	FS		CS		HC		Differences
*n*; (% male)	52; 55.8		50; 52		60; 41.7		FS–CS *p* > 0.05
							FS–HC *p* > 0.05
							CS–HC *p* > 0.05
Age (years)	$\bar{x}$ **(Me)**	**SD**	$\bar{x}$ **(Me)**	**SD**	$\bar{x}$ **(Me)**	**SD**	
	22.67 (21.0) *	5.12	41.52 (40.5) *	11.31	31.05 *	10.62	FS–CS *p* < 0.001
							FS–HC *p* < 0.001
							CS–HC *p* < 0.001
BMI (kg/m^2^)	22.6 (21.6) *	3.80	26.4 (26.0) *	4.72	25.0 (24.2) *	5.39	FS–CS *p* < 0.001
							FS–HC *p* = 0.042
							CS–HC *p* = 0.114
Number of cigarettes per day	5.71 (0)	8.29	5.86 (0)	8.72	1.78 (0)	4.92	FS–CS *p* = 1.000
							FS–HC *p* = 0.045
							CS–HC *p* = 0.006
Gluten intake (mg/d)	17.13 (16.76)	8.47	18.77 (18.98) *	7.01	13.98 (14.16) *	7.30	FS–CS *p* = 0.522
							FS–HC *p* = 0.076
							CS–HC *p* = 0.003
Gastric symptoms (points: VAS-IBS)	2.83 (2.0) *	2.84	1.38 (0) *	2.12	2.88 (2.0) *	3.07	FS–CS *p* = 0.006
							FS–HC *p* = 1.000
							CS–HC *p* = 0.006
Duration of illness (months)	9.90 (5.0) *	9.87	239.72 (228) *	135.09	NA		FS–CS *p* < 0.001
Number of hospitalizations	1.64 (1) *	1.46	5.8 (5) *	4.22			FS–CS *p* < 0.001
PANSS total (points)	95.85 (100) *	20.85	73.66 (72.5) *	18.54			FS–CS *p* < 0.001
Dose of olanzapine equivalents (mg)	4.65 (0) *	7.12	17.72 (18.0) *	8.47			FS–CS *p* < 0.001

VAS-IBS—Visual Analogue Scale for Irritable Bowel Syndrome; FS—first episode of schizophrenia; CS—chronic schizophrenia; HC—healthy controls; BMI—body mass index; PANSS—Positive and Negative Symptoms Scale; $\bar{x}$ —mean; Me—median; SD—standard deviation; NA—not applicable; NS—not significant; *—statistically significant according to Kruskal–Wallis test after Bonferroni correction.

**Table 2:** Differences in serum concentrations of gluten sensitivity, gut permeability, and inflammatory markers among the examined groups.

Group	$\bar{x}$ **(Me)**	SD	Kruskal–Wallis Test	Post-Hoc Analysis
**AGA IgA U/mL**				
FS	7.32 (6.03)	4.49	H = 6.22 *p = 0.045*	CS > HC
CS	10.65 (6.61)	18.11		
HC	6.08 (5.77)	2.97		
**AGA IgG U/mL**				
FS	17.01 (4.97)	42.21	H = 3.93 *p = 0.14*	NS
CS	9.22 (3.26)	16.69		
HC	6.72 (4.92)	9.18		
**anti-tTG IgA U/mL**				
FS	16.43 (15.55)	8.18	H = 2.55 *p = 0.38*	NS
CS	20.06 (16.78)	11.76		
HC	18.86 (16.19)	9.99		
**anti-DGP IgG U/mL**				
FS	22.92 (8.72)	43.68	H = 4.40 *p = 0.111*	NS
CS	12.09 (6.52)	15.68		
HC	10.95 (7.18)	18.06		
**ASCA IgG U/mL**				
FS	3.27 (0.69)	5.04	H = 9.07 *p = 0.011*	FS < HC
CS	5.41 (2.48)	7.97		
HC	5.15 (3.89)	4.72		
**sCD14 pg/mL**				
FS	1632.11 (1651.63)	387.58	H = 13.84 *p = 0.001*	CS > HC
CS	1960.94 (1784.48)	766.22		
HC	1487.43 (1563.88)	452.62		
**hsCRP µg/mL**				
FS	1.54 (0.50)	2.40	H = 20.26 *p < 0.001*	CS > FS CS > HC
CS	3.13 (1.31)	3.30		
HC	0.99 (0.43)	1.39		
**IL-6 pg/mL**				
FS	4.49 (3.64)	4.05	H = 7.50 *p = 0.023*	CS > HC
CS	6.49 (5.05)	6.79		
HC	4.56 (3.02)	6.21		

FS—first episode of schizophrenia; CS—chronic schizophrenia; HC—healthy controls; BMI—body mass index; $\bar{x}$ —mean; Me—median; SD—standard deviation; AGA IgA—antigliadin antibodies IgA; AGA IgG—antigliadin antibodies IgG; anti-tTG_2_ IgA—antibodies against tissue transglutaminase 2 IgA; anti-DGP IgG—anti-deamidated gliadin peptides IgG; ASCA—anti-*Saccharomyces cerevisiae* antibody; sCD14—soluble CD14; CRP—high-sensitivity C-reactive protein; IL-6—interleukin-6.

**Table 3:** The proportion of individuals with elevated concentrations of gluten sensitivity and inflammatory markers across the examined groups.

	FS (%)	CS (%)	HC (%)	Chi-Square Test
AGA IgA	11.54	26	5	*p* = 0.005
AGA IgG	30.77	20	10	*p* = 0.023
anti-tTG_2_ IgA	1.92	2	0	NS
anti-DGP IgG	5.77	2	2	NS
ASCA	0	6	0	*p* = 0.033
sCD14	0	4	0	NS
hsCRP	17.31	36	6.67	*p* < 0.001
IL-6	84.62	90	91.67	NS

AGA IgA—antigliadin antibodies IgA; AGA IgG—antigliadin antibodies IgG; anti-tTG_2_ IgA—antibodies against tissue transglutaminase 2 IgA; anti-DGP IgG—anti-deamidated gliadin peptides IgG; ASCA—anti-*Saccharomyces cerevisiae* antibody; sCD14—soluble CD14; CRP—high-sensitivity C-reactive protein; IL-6—interleukin-6.

## Supplementary Materials

The following are available online at https://www.mdpi.com/2077-0383/9/11/3707/s1. Table S1: The relationship between gluten sensitivity and inflammation, Table S2: The relationship between gluten sensitivity, inflammation, and characteristics of the examined population.
