# Supplementary material for: The Differences between Gluten Sensitivity, Intestinal Biomarkers and Immune Biomarkers in Patients with First-Episode and Chronic Schizophrenia
Source: J Clin Med. 2020 Nov 18;9(11):3707. doi: 10.3390/jcm9113707 (PMC7699286; doi:10.3390/jcm9113707)
Supplement: Supplementary file 1 [file jcm-09-03707-s001.pdf]

Journal of
Clinical Medicine

MDPI

Table 1. The relationship between gluten sensitivity and inflammation.

	ASCA			sCD14			AGA IgA		
	**FS**	**CS**	**HC**	**FS**	**CS**	**HC**	**FS**	**CS**	**HC**
AGA IgG	0.340	0.294	NS	NS	NS	NS	0.299	NS	NS
hsCRP	NS	NS	NS	0.381	0.299	NS	NS	NS	NS

AGA IgA—antigliadin antibodies IgA; AGA IgG—antigliadin antibodies IgG; ASCA—anti-Saccharomyces Cerevisiae antibody; sCD14—soluble CD14; hsCRP—high-sensitivity C-reactive protein; FS—first episode schizophrenia; CS—chronic schizophrenia; HC—healthy control.

Table 2. The relationship between gluten sensitivity, inflammation and characteristics of examined population.

	AGA IgG			AGA IgA			Anti-tTG2 IgA			ASCA			hsCRP			IL-6		
	**FS**	**CS**	**HC**	**FS**	**CS**	**HC**	**FS**	**CS**	**HC**	**FS**	**CS**	**HC**	**FS**	**CS**	**HC**	**FS**	**CS**	**HC**
Age	NS	-0.419	NS	NS	NS	NS	NS	NS	NS	NS	-0.401	NS	0.276	NS	0.387	NS	NS	0.278
BMI	NS	NS	NS	NS	NS	NS	NS	NS	NS	NS	NS	NS	NS	0.412	0.560	NS	NS	NS
Gastrointestinal symptoms	0.322	NS	NS	NS	NS	NS	NS	NS	NS	NS	NS	NS	NS	NS	0.275	NS	NS	NS
Number of meals per day	NS	NS	NS	NS	NS	NS	NS	-0.320	NS	NS	NS	NS	NS	NS	NS	NS	NS	NS
Number of cigarettes smoked per day	NS	NS	NS	NS	NS	NS	NS	NS	NS	NS	NS	NS	NS	NS	NS	-0.368	NS	0.379
Duration of illness	NS	-0.394	NS	-0.289	NS	NS	NS	NS	NS	NS	-0.387	NS	NS	NS	NS	0.310	NS	NS

AGA IgA—antigliadin antibodies IgA; AGA IgG—antigliadin antibodies IgG; anti-tTG$_2$ IgA—antibodies against tissue transglutaminase 2 IgA; ASCA—anti-Saccharomyces Cerevisiae antibody; sCD14—soluble CD14; hsCRP—high-sensitivity C-reactive protein; IL-6—interleukin-6; BMI—Body Mass Index; FS—first episode schizophrenia; CS—chronic schizophrenia; HC—healthy control

© 2020 by the authors. Licensee MDPI, Basel, Switzerland. This article is an open access article distributed under the terms and conditions of the Creative Commons Attribution (CC BY) license (http://creativecommons.org/licenses/by/4.0/).
